# Congo Red Decolorization and Detoxification by* Aspergillus niger*: Removal Mechanisms and Dye Degradation Pathway

**DOI:** 10.1155/2018/3049686

**Published:** 2018-08-06

**Authors:** Nedra Asses, Lamia Ayed, Neila Hkiri, Moktar Hamdi

**Affiliations:** LR-Microbial Ecology and Technology, INSAT, University of Carthage, Tunis, Tunisia

## Abstract

Congo red is one of the best known and used azo dyes which has two azo bonds (-N=N-) chromophore in its molecular structure. Its structural stability makes it highly toxic and resistant to biodegradation. The objective of this study was to assess the congo red biodegradation and detoxification by* Aspergillus niger*. The effects of pH, initial dye concentration, temperature, and shaking speed on the decolorization rate and enzymes production were studied. The maximum decolorization was correlated with lignin peroxidase and manganese peroxidase production. Above 97% were obtained when 2 g mycelia were incubated at pH 5, in presence of 200 mg/L of dye during 6 days at 28°C and under 120 to 150 rpm shaking speed. The degraded metabolites were characterized by using LC-MS/MS analyses and the biodegradation mechanism was also studied. Congo red bioconversion formed degradation metabolites mainly by peroxidases activities, i.e., the sodium naphthalene sulfonate (m/z = 227) and the cycloheptadienylium (m/z = 91). Phytotoxicity and microtoxicity tests confirmed that degradation metabolites were less toxic than original dye.

## 1. Introduction

Water is necessary to sustaining life on earth. However, available water represents less than 1% of the total volume of fresh water on earth [1]. However, pollution reduces its availability for human use. The water pollutants are usually generated by industries and can be divided into various classes. Every class has its own specific dangers.

The textile industry is one of the most polluting industries of clean water. In fact, during the manufacturing processes, a large percentage of the synthetic dye does not bind and is lost in wastewaters, which are usually discharged untreated. Congo red is the most common dyes that can be found in textile industry. It is a benzidine based anionic diazo dye [2]. This dye is known to be metabolized into benzidine, which is a human carcinogen and mutagen; that is why it is banned in many countries.

Various chemical and physical methods of these colored waste waters have been proposed in the last few decades such as coagulation–flocculation, oxidation, and electrochemical methods [3]. Nowadays scientists work on the implementation of innovative processes to treat these recalcitrant compounds. Among the most recent treatments is the advanced oxidation process (AOP), which allows mineralization of toxic organic molecules through formation of extremely reactive and nonselective radicals such as hydroxyl radicals. However, AOP have many disadvantages, such as high-energy costs and sometimes formation of toxic by-products [4].

However, bioprocessing can be considered as a preferred option to overcome these disadvantages because it is cost saving and environmentally friendly. Biological treatments can be used to degrade and/or to adsorb azo dyes contaminants [5]. The most efficient microorganisms to break down colored pollutants so far reported are white-rot fungi. These comprise mostly basidiomycetous fungi, which are capable of extensive aerobic lignin degradation and mineralization. This is possible through several extracellular lignin-degrading enzymes [6], such as lignin peroxidase, manganese-dependent peroxidase, and laccase. A previous report showed dye degradation potential of* Aspergillus niger* [7]. A total decolorization of Procion Red MX-5B by* Aspergillus niger* was obtained after 336 h of treatment. On the other hand, the enzymatic cleavage of azo dyes leads to the formation of toxic products, mainly amines. Therefore, it is important to identify and evaluate toxicity of degradation products [8].

The aim of the present work was to study the* Aspergillus niger* potential for detoxification and decolorization of CR dye. The enzymes involved in the decolorization process were identified and the effects of various parameters (pH, temperature, initial dye concentration, and shaking speed) on dye decolorization and enzymes production were investigated. The degraded metabolites were characterized by using LC-MS/MS analyses. The study also aimed to assess the toxicity of the metabolites formed after the degradation of CR dye by this fungus.

## 2. Materials and Methods

### 2.1. Fungal Strain and Biomass Generation

*Aspergillus niger *was isolated from processing wastewater [9]. The fungal strain was maintained on Potato Dextrose Agar (Merck) at room at 28 ± 2°C. To generate biomass, three mycelia plugs (0.7 cm diameter) taken from the edge of the colony were transferred into 100 ml synthetic nutrient broth medium containing (g. L^−1^) glucose 10 g, yeast extracts 1 g, and peptone 2 g (Scharlau). The pH was adjusted to 6.0 ± 0.2 and the culture was incubated for 8 days at 30°C. The fungal biomass was homogenized, filtered through Whatman filter paper No. 1, and washed with sterile distilled water. This freshly prepared biomass was used for dye biodegradation experiments.

### 2.2. Condition Optimization for CR Decolorization

The fungus ability to decolorize the CR dye (Sigma Aldrich) under different conditions was investigated using decolorization rate as the index. The influence of pH and temperature on decolorization was studied in presence of 200 mg. L^−1^ CR under pH values ranging from 3 to 10 and temperature ranging from 15 to 45°C. The agitation effect was studied under different speed shacking conditions (0, 50, 100, 150, and 200 rpm). The influence of initial dye concentration was tested using 100, 250, 500, and 1000 mg. L^−1^. In all cultures, 2g of fungal fresh biomass was used to inoculate 100 mL synthetic nutrient broth. Cultures were incubated at 30°C, for 6 days. The supernatant was used for color reduction measurements and peroxidases activities.

### 2.3. Biodegradation and Biosorption Treatments

The CR biodegradation treatment was performed with an aqueous solution inoculated with 20g L^−1^ of fresh fungal biomass. In 250 mL Erlenmeyer containing 100 mL of synthetic broth medium was supplemented with 200 mg. L^−1^ CR dye at pH 6.0 ± 0.2. After inoculation, cultures were placed in a rotary shaker at 150 rpm and 30°C for 10 days. Noninoculated dye solution was designated as negative control. At regular intervals, a sample from the broth medium was collected and centrifuged at 5000 rpm for 15 min to remove the fungal mycelium. The supernatant was used for pH, color reduction measurements, and enzyme activities assay. The fungal biomass was determined by measuring the dry weight of the pellets suspensions washed twice with distilled water. All cultures were performed in triplicate, and the results are the average.

CR biosorption treatment was conducted with dye aqueous solution in 250 mL Erlenmeyer containing 100 mL broth medium and 200 mg. L^−1^ CR dye inoculated by 2g of autoclaved fresh fungal biomass of* A. niger *(inactivated biomass). Flasks were incubated at 30°C for 48 h under stirred conditions (120 rpm). Noninoculated dye solution was designated as negative control.

### 2.4. Enzymatic Assay

Lignin peroxidase (LiP) activity was determined using veratryl alcohol as a substrate [10]. The assay mixture contained 2 mM veratryl alcohol and 0.4 mM H_2_O_2_ in 50 mM sodium tartrate buffer, pH 2.5. Oxidation of veratryl alcohol was followed by measuring the increase in absorbance at 310 nm because of the formation of veratraldehyde from *ε*_310_ = 9300 M^−1^ cm^−1^.

Manganese peroxidase (MnP) activity was determined using MnSO4 as a substrate [11]. The assay mixture contained 0.5 mM MnSO4 and 0.5 mM H_2_O_2_ in 50 mM sodium malonate buffer, pH 4.5. Oxidation of Mn^2+^ was followed by measuring the increase in absorbance at 270 nm due to the formation of Mn^3+^-malonate from *ε*_270_=11590 M^−1^ cm^−1^.

Laccase activity was measured spectrophotometrically with a Genesys 5 spectrophotometer using 1 *μ*mol 2, 2-azino–bis (3-ethylbenzothiazoline-6-sulfonate) (ABTS) as a substrate. One unit of enzyme activity was defined as of ABTS oxidized per minute at 25°C (*ε*_420_= 29 300 M^−1^ cm^−1^) [12]. All enzyme activity was expressed as international units (IU).

### 2.5. UV-Visible and FTIR Analyses

Color reduction was assayed by absorbance measurement at *λ*_max_ =495nm nm using a Jenway 3540 UV/VIS spectrophotometer. After culture centrifugation at 5000 rpm for 15 min, the supernatants were analyzed by measuring the absorbance differences. Decolorization rate was determined according to the following formulation:

(1) $$\begin{array}{c} \text{Decolorization rate}\left(\;\%\;\right)=\frac{\left(A_{0}-A_{1}\right)}{A_{0}}\times 100 \end{array}$$

A_0_ is the dye absorbance before decolorization and A_1_ is the dye absorbance after decolorization.

The decolorization analysis of the biodegraded and crude CR solution was performed by the change in the absorption spectrum in the wavelength range of 200-800 nm region using a quartz cuvette with an optical path of 5nn.

FTIR analysis of lyophilized fungal biomass was monitored on a Thermo Scientific IR 200 FT-IR spectrophotometer. The FTIR spectra were then recorded between 4000 and 400 cm^−1^, at a rate of 16 nm/s.

### 2.6. LC-MS/MS Analyses of Transformed Metabolites

CR metabolites were analyzed on a LC-MS/MS with an electrospray ionization- (ESI-) interface (Agilent Technologies, USA) equipped with C18 waters column (4.6 mm-250 mm; particle size 5um). Isocratic elution was performed with mobile phase of methanol: water (90:10 v/v) at a flow rate of 0.7 mL/min.

### 2.7. Toxicity Assay

#### 2.7.1. Phytotoxicity Assay

The phytotoxicity assays were performed to assess the toxicity of dye before and after degradation. The experiment was conducted using* Zea mais* and* Solanum lycopersicum* seeds. 10 seeds were wetted (3mL per day) with dye solution (200 mg. L^−1^) or treated CR solution in separated Petri dishes. The control groups were treated with distilled water. All samples were incubated at the same environmental conditions and repeated three times. Percent of germination and length of shoot and root were recorded after 7 days.

#### 2.7.2. Microtoxicity Assay

*Bacillus cereus* ATCC 11778 and* Escherichia coli* ATCC 10536 strains were used for toxicity evaluation of the untreated and treated RC solution by* Aspergillus niger*. The two strains growths were studied on nutrient broth (NB) medium as control and on NB medium supplemented with 200 mg L^−1^ of CR before and after biodegradation. Incubation temperature was 30°C and 37°C for* B. cereus* and* E. coli*, respectively. The bacterial growth was assessed by OD at 600 nm recorded at 1 h interval during 8 h [13].

### 2.8. Statistical Analysis

An analysis of variance (a one-way ANOVA) was conducted by employing performed (SPSS) version 16.0 software. SAS 9.0 software was used for all statistical analysis with multiple comparison tests. Effects were considered significant when the P value was < 0.05.

## 3. Results and Discussion

### 3.1. Process Parameters' Optimization for Congo Red Decolorization by* A. niger*

**Figure 1:**
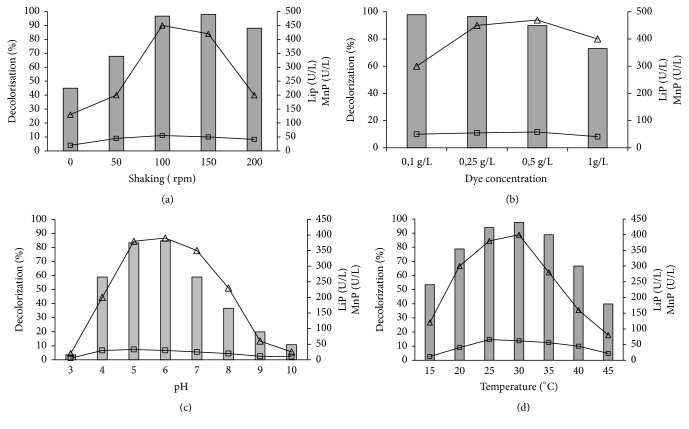
Effect of initial dye shaking (a), dye concentration (b), pH (c), and temperature (d) on CR decolorization efficiency (Gray colored bars), LiP (□), and MnP activities (∆).

CR decolorization efficiency and enzyme production (LiP and MnP) by* A. niger* were investigated by different process parameters. Effects of speed shaking, pH, temperature, and initial dye concentration are shown in Figure 1. The effect of shaking speed was found to be highly significant (*p* < 0.01) on CR decolorization. When the speed increases from 0 to 150 rpm, the decolorization efficiency increases from 45% to 98% after six days of culture, indicating that shaking increases the mixing of the oxygen present in the medium, thus helping* Aspergillus niger* growth (Figure 1(a)). The same results were obtained by Kumar et al. [14], who found that shaking was beneficial for achieving maximum dye decolorization of brilliant green by* Aspergillus sp*. as a result of better oxygen transfer and nutrient distribution through the medium. However, a decline was observed beyond a speed of 150 rpm, which can be explained by pellet formation decrease in spite of the high biomass produced. The maximum LiP and MnP activities were obtained at a speed of 100 rpm (54 and 452 U.L^−1^, respectively). The differences between enzymatic activities recorded between 100 and 150 rpm are not significant (P > 0.05). When the speed increased from 150 to 200 rpm, the MnP activity decreased but LiP activity remained almost constant. Mahmoud et al. [15] reported an increase in the decolorization rate of direct red azo dye by* A. niger* with increasing the agitation speed. The maximum removal efficiency was recorded at agitation speed of 250 rpm, after which no significant increase was noticed. On the contrary, some authors noticed decolorization decreased under shaking condition due to the competition between azo dyes and oxygen for reduced electron carriers [16].

CR decolorization is also affected by medium pH.* A. niger* showed high CR removal (85%) and enzyme activities under slightly acidic pH varying from 5 to 6 (differences are not significant P > 0.05 ) (Figure 1(b)). In fact, MnP activity notably dropped at high pH values (pH > 7), which can be due to enzymes stability. This enzyme may be stable only at acidic pH. Michaels and Lewis 1986 showed that medium pH is one of the critical environmental factors that affects azo biodegradation. Kumar et al. 2011 obtained methyl violet biodegradation by* Aspergillus *sp. only in slightly acidic conditions (pH 5.5) and that the decolorization was inhibited at a pH higher than 6.5.

From Figure 1(c), it can be deduced that a temperature around 30°C is the most favorable temperature to carry out this decolorization and a significant decrease was observed at higher temperature. This can be explained by cell viability reduction at high temperature or to the inactivation of the enzymes responsible for RC decolorization. Parshetti et al. [17] showed that optimum temperature for dye decolorization for most fungi varied between 25 and 35°C.

Figure 1(d) shows that CR removal was slightly inhibited at 0.5gL-1, and the best result was found with 0.25 g.L^−1^ with a decolorization rate of 96% and enzyme production of 56 and 470 U.L^−1^ for LiP and MnP, respectively. When the initial dye concentration increased to 1g.L-1, a significant reduction in the decolorization rate was observed (73%), which can be due to the dye toxicity at high concentrations [18]. Same result was reported by Parshetti et al. [17], who showed that reactive blue-25 at high concentration affected the decolorization performance of* Aspergillus ochraceus*.

### 3.2. Time Effect on CR Decolorization and Enzymatic Activities

**Figure 2:**
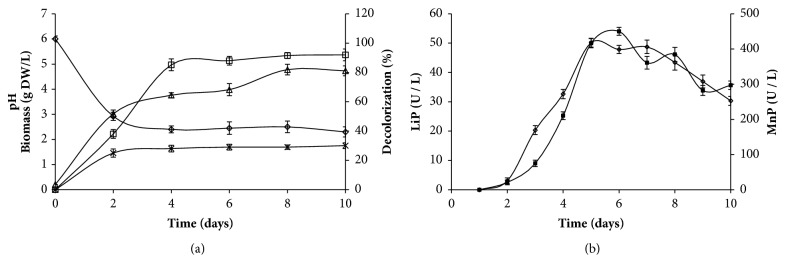
Time course of pH (◊), Biomass (△), and decolorization (□: with activated* A. niger*, x: with inactivated* A. niger*) (a); LiP (▲) and MnP (■) (b) during azo dye CR treatment by* A. niger. *The error bars represent the standard deviation of measurements for 3 samples.

Optimal conditions previously set up were used to study the biodegradation and the adsorption ability of* A. niger*. RC removal and enzymatic activities were conducted with 2 g of fresh activated or inactivated mycelia, inoculated into 100 mL of synthetic media containing 250 mg. L-1 of CR dye, and incubated at 30°C. Decolorization, biomass production, pH, LiP, and MnP activities were monitored during 6 days (Figure 2).

The biomass increased and pH decreased the first six days and then stabilized. The maximum mycelium dry weight obtained was 6.4 g.L^−1^.* A. niger *growth induced a significant decolorization that reached 97% after 6 days of incubation. However, inactivated cells led to 27% color removal during the same period (Figure 2(a)). Then, the decolorization could be attributed to both biodegradation and dye adsorption on the fungal mycelium. Therefore, 97% of decolorization is the result of two-process combination, the enzymatic activity responsible for the molecule degradation and adsorption phenomena. In fact, 1g of fresh biomass can eliminate 27% of CR dye by adsorption mechanism and 70% by enzymatic biodegradation.

Time course for LiP and MnP produced by* A. niger* was achieved in order to evaluate the maximum enzyme activity during CR removal by* A. niger* (Figure 2(b)). LiP activity was detected after 2 days and reached a maximum at the 5th of the culture. However, maximum MnP activity was obtained in the 6th day. The highest MnP and LiP activities were 53.2 ± 1.41 UI.L^−1^ and 450.13 ± 11 UI.L^−1^, respectively. However, laccase activity was not detected. Since the MnP activity level produced was much higher than the LiP activity, MnP seemed to play the most important role in the decolorization. The presence of ligninolytic extracellular enzymes in culture supports biological decolorization alongside the nonbiological color removal by adsorption [19]. Several studies have shown that ligninolytic fungal enzymes are efficient for dye decolorization [20, 21]. Parshetti et al. [17] have shown the presence of lignin peroxidase, laccase, and tyrosinase produced by* Aspergillus ochraceus* during reactive blue 25 decolorization.

### 3.3. CR Color Removal By* A. niger*

**Figure 3:**
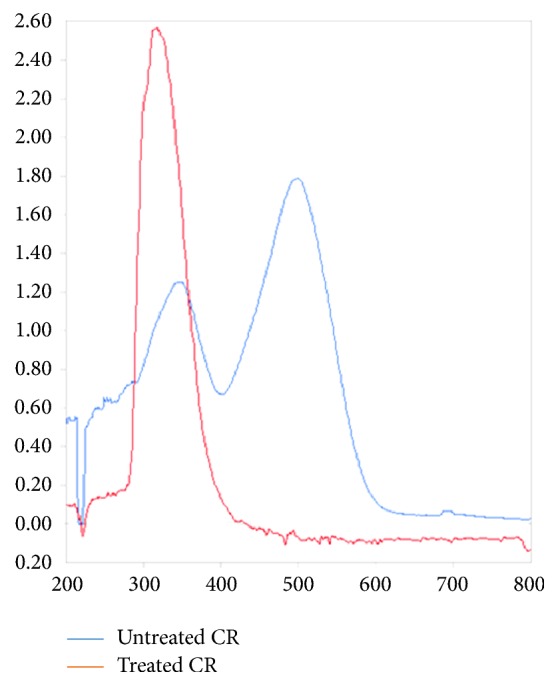
Changes in UV-visible absorption spectrum of CR (200 mg.L-1) before and after CR decolorization by* A. niger* treatment at shaking condition (200 mg. L^−1^ CR dye at pH 6.0 ± 0.2 shaked at 150 rpm incubated at 30°C for 8 days).

CR color removal was confirmed by UV-visible analysis (200-800 nm) (Figure 3). CR UV-Vis scanning showed two peaks. One extended at 340 nm, which is due to the interaction between aromatic hydrocarbon or polycyclic aromatic hydrocarbon groups and other chromophore and another at 495 nm, which had a relation to azo double bond and large conjugated system for the whole dye molecule. After 96 h treatment, a decrease in the major peak intensity at 495nm was observed. It indicated that dye structure especially that of chromophore was transformed during* A. niger* treatment. However, the absorbance at 340 nm was increased which evinced that interaction of the aromatic hydrocarbons or polycyclic aromatic hydrocarbon groups and some chromophore were partly destroyed, or new aromatic compounds might appear [22]. These changes suggested that* A. niger *is able to transform the CR dye to other compounds. This result suggested the chromophore group's breakdown. Wang et al. [23] reported that the degradation of the aromatic hydrocarbon or polycyclic aromatic hydrocarbon groups completely seemed to be more difficult than the destruction of the azo double bond and the large conjugated system.

### 3.4. CR Adsorption on* A. niger* Biomass

**Figure 4:**
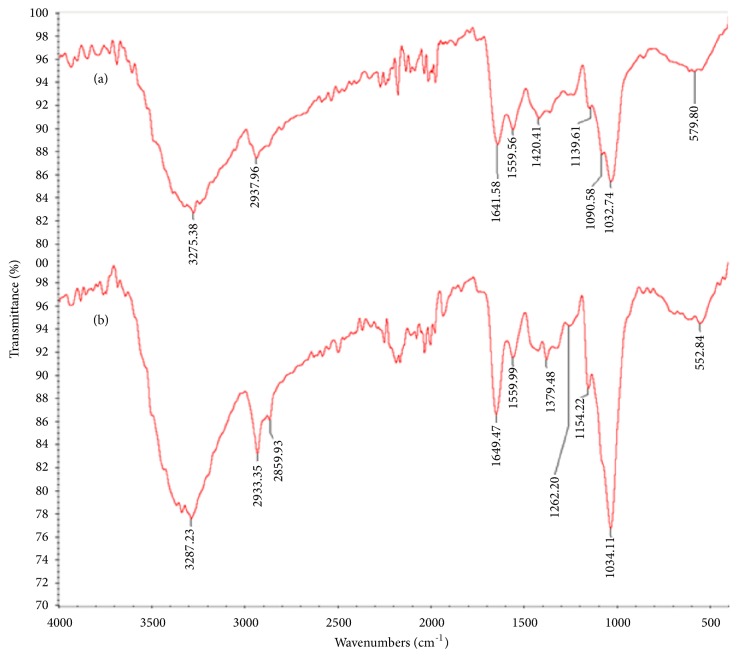
FTIR spectra of inactive* A. niger* before (a) and after biosorption of CR dye (b) (100 mL broth medium and 200 mg. L^−1^ CR dye inoculated by 2g of autoclaved fresh fungal biomass of* A. niger *(inactivated biomass) incubated at 30°C for 48 h under stirred conditions (120 rpm)).

In order to elucidate the nature of the functional groups responsible for the biosorption, FTIR analysis of the lyophilized biomass was carried out before and after incubation in CR solution (200 mg.L-1) (Figures 4(a) and 4(b)). The FTIR spectra of* A. niger* biomass before treatment showed the characteristic band at 3275 cm−1, which is attributed to O–H bending vibrations. The peak at 2937 cm−1 corresponds to asymmetric and symmetric stretching of the C- H bond of -CH2 group. The band at 1641 cm−1 is due to the bending of N–H groups of chitin on the cell wall structure of fungal pellets [9]. The peaks around 1559 cm−1 indicated the presence of amide which resulted from NH deformation. The bands at 1420 cm−1, 1139 cm-1, and 1090 cm−1 are representing -CH3 wagging (umbrella deformation), symmetric –SO3 stretching, and C–OH stretching vibrations, respectively, which were due to several functional groups present on the fungal cell walls [22]. The peak at 579 cm−1 is corresponding to C–O bending vibrations. The adsorption of CR on the fungal biomass induced an increase in some peaks intensity, in particular, those around 3287, 2933, 1649, 1154, and 1034cm-1. An appearance of new peaks at 2859, 1379, 1262, and 1154 cm-1 was due to introduction of new functionalities on the surface of biosorbent which confirmed the CR adsorption on fungal biomass. Similar FTIR results were observed for the phenolic compounds' biosorption on various fungus biomass [23].

### 3.5. Bioconversion of CR by* A. niger*

**Figure 5:**
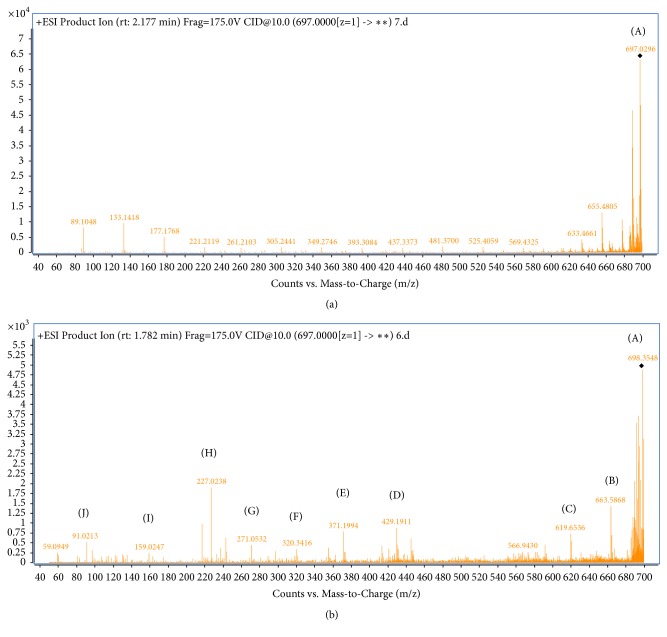
LC-MS/MS spectrum of crude CR (a) and degraded product formed during the CR decolorization process by* A. niger *(b) (200 mg. L^−1^ CR dye at pH 6.0 ± 0.2 shaked at 150 rpm incubated at 30°C for 8 days).

**Figure 6:**
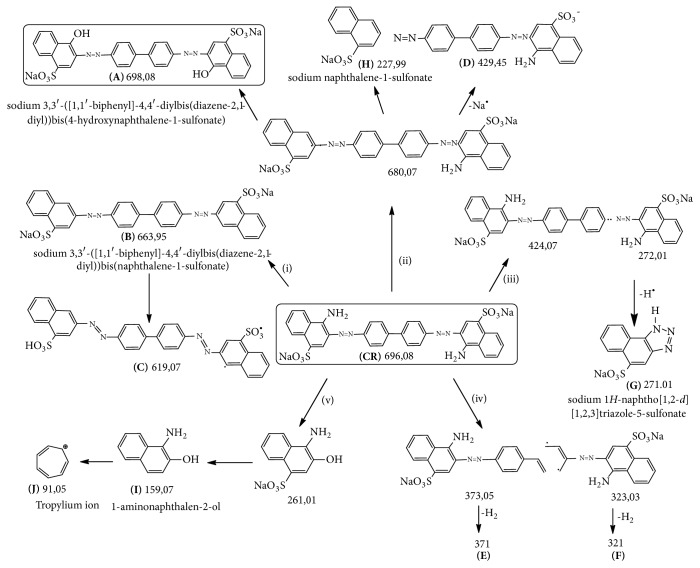
Proposed biodegradation pathway of CR using* A. niger*, with the identification of different degradation intermediates by LC-MS/MS.

LC-MS/MS spectra of both treated and nontreated CR dye solution display different patterns the major several compounds obtained after CR decomposition at different m/z ratios confirming the CR biodegradation by* A. niger* (Figure 5). According to LC-MS/MS spectrum, the possible degradation pathway for CR dye is showed in Figure 6. The degradation of the CR dye may occur via the following steps: (i) the simultaneous total deamination and oxygenation of (CR) forming the compound (A) with m/z value of 698. When the total deamination is followed by the loss of both sodium atoms, the degraded products (B) and (C) with m/z values of 663 and 619, respectively, are formed. (ii) The partial deamination of (CR) and the peroxidase asymmetric cleavage of C-N bond between the aromatic ring and the azo group with the loss of a sodium atom afford the intermediates (H) and (D) with m/z values of 227 and 429, respectively. (iii) The peroxidase asymmetric cleavage of C-N bond followed by deprotonation leaded to the intermediate (G) with m/z value of 271. (iv) The benzene ring opening and dehydrogenation formed intermediates (E) and (F) with m/z values of 371 and 321, respectively. (v) The peroxidase cleavage produces also low molecular weight of stable degraded products, the sodium naphthalene sulfonate (I) (m/z = 227) and the cycloheptadienylium (J) (m/z=91). In addition to LiP and MnP as degrading agents, Figures 5 and 6 showed that the most abundant intermediates of degradation (A, B, C, H, and D) resulted from deamination of the CR dye. This is in agreement with the literature which reported the GlcN6P desaminase role of* A. niger* [24].

### 3.6. Phytotoxicity Analysis

Phytotoxicity analysis revealed the toxicity of CR and its products to* Zea mais* and* Solanum lycopersicum *seeds (Table 1). Compared to water treatment, CR before degradation reduced significantly the germination rate, shoot, and root length of both* Zea mais* and* Solanum lycopersicum* (p < 0.01). Nevertheless, the metabolites generated after the CR biodegradation are less toxic than the crude dye. In fact, there is no difference between the shoot and root length of both* Zea mais* and* Solanum lycopersicum* treated with treated CR and with water (differences were not significant with P > 0.05). Babu et al. [5] showed that the degradation metabolites of CR are comparatively less toxic than the crude CR to* Artemia franciscana*. Therefore, in addition to its degradation, the CR dye is detoxified by* A. niger* indicating the degradation of the amines in the solution. This result is in accordance with the deaminating effect of GlcN6P deaminase in* A. niger *observed in LC-MS/MS data.

### 3.7. Microtoxicity Analysis

**Figure 7:**
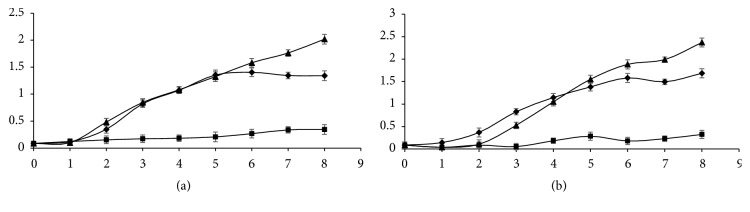
Kinetics growth of* Bacillus cereus ATCC 11778 (a) and Escherichia coli ATCC 10536 (b) *on nutrient broth, CR (200 mg.L^−1^), and its transformation metabolites. The error bars represent the standard deviation of measurements for 3 samples.

Microbial toxicity of crude CR solution (200 mg.L-1) and treated one was tested against* B. cereus* ATCC 11778 and* E. coli* ATCC 10536 (Figure 7). Inhibition was obtained with untreated CR dye solution. In fact, after 8 hours of incubation* B. cereus* and* E. coli* density remained very lower in the media containing crude CR than the control ones, with a significant difference (p < 0.05), while an improvement of bacterial growth was observed in the media containing treated dye. Density values of* B. cereus* and* E. coli* were 1.34 and 1.68, respectively, on treated CR and 2.019 and 2.37, respectively, on nutrient broth. Therefore, the CR degradation seems to detoxify the azo dye.

## 4. Conclusion

This study revealed that the CR was successfully decolored and biodegraded by* Aspergillus niger.* High decolorization efficiency (97%) was obtained after six days of culture. 1g of fresh biomass can eliminate 27% of CR dye by adsorption mechanism and 70% by enzymatic biodegradation. This degradation is due to the combined action of three enzymes LiP, MnP, and probably deaminase. UV-Vis, FTIR, and LC-MS/MS analysis as well as phytotoxicity and microtoxicity tests have proven the effective role of degrading and detoxifying CR dye by* A*.* niger*. This biological process is recommendable for further development as a potential technology for wastewater treatment.

## Figures and Tables

**Table 1:** Phytotoxicity study of CR before and after biodegradation by *A. niger on Zea mais* and *Solanum lycopersicum*.

Parameters	*Zea mais *			*Solanum lycopersicum*		
	Water	Congo red	Transformation intermediates	Water	Congo red	Transformation intermediates
Germination (%)	91± 2.02	60 ± 3.52 (*∗*)	82 ± 3.05 (Ns)	88± 2.08	60± 3.78 (*∗*)	81±2.72 (Ns)
Shoot length (cm)	12.2±1.02	6.5±0.36 (*∗*)	10.9±0.52 (Ns)	5.16±0.44	1.8±0.15 (*∗∗*)	4.66±0.44 (Ns)
Root length (cm)	4.26±0.37	1.7±0.14 (*∗∗*)	3.16±0.21 (Ns)	3.13±0.12	2.06±0.17 (*∗*)	3.2±0.15 (Ns)

Ns: differences are not significant; *∗*: differences are significant at P ≤ 0.05, *∗∗*: differences are significant at P ≤ 0.01 according to ANOVA statistical analysis.

## Data Availability

The data used to support the findings of this study are available from the corresponding author upon request.
